# The Duties of Practitioners in Relation to Their Professional Services to Each Other, to Their Families, Widows and Children

**Published:** 1882-06-20

**Authors:** 


**The Duties of Practitioners in Relation to their Professional Services to Each Other, to their Families, Widows and Children.**—All legitimate practitioners of medicine, their wives, and children while under the paternal care, are entitled (not as a matter of right, but by professional courtesy) to the reasonable and gratuitous services—railway and like expenses excepted—of the faculty resident in their immediate neighborhood, whose assistance may be desired. In the case, also, of near relatives who are more or less dependent upon a professional brother (other than wealthy), it will likewise be well, at his request, to forego or to modify the usual fee. On the other hand, a son or daughter altogether independent of the father, or the widow and children of a practitioner left in affluent circumstances, should be charged as ordinary patients, unless feelings of friendship or other special reasons render the attendant practitioner averse to professional remuneration. In such case the rule need not apply. Moreover, if a wealthy member of the faculty seeks professional advice, and courteously urges the acceptance of a fee, it should not be declined; for no pecuniary obligation ought to be imposed on the debtor which the debtee himself would not wish to incur.—Proposed amendment to Code of Medical Ethics, British Medical Journal, 1882, p. 480.—*Medical Times.*

